# Real-time location system-based asset tracking in the healthcare field: lessons learned from a feasibility study

**DOI:** 10.1186/s12911-018-0656-0

**Published:** 2018-09-10

**Authors:** Sooyoung Yoo, Seok Kim, Eunhye Kim, Eunja Jung, Kee-Hyuck Lee, Hee Hwang

**Affiliations:** 1 Office of eHealth Research and Businesses, Seoul National University Bundang Hospital, 166, Gumi-ro, Bundang-gu, Seongnam-si, 436-707 South Korea; 2 Nursing Department, Seoul National University Bundang Hospital, 166, Gumi-ro, Bundang-gu, Seongnam-si, 436-707 South Korea

**Keywords:** Asset tracking, Efficiency, Healthcare, Real-time locating system, System implementation

## Abstract

**Background:**

Numerous hospitals and organizations have recently endeavored to study the effects of real-time location systems. However, their experiences of system adoption or pilot testing via implementation were not shared with others or evaluated in a real environment. Therefore, we aimed to share our experiences and insight regarding a real-time location system, obtained via the implementation and operation of a real-time asset tracking system based on Bluetooth Low Energy/WiFi in a tertiary care hospital, which can be used to improve hospital efficiency and nursing workflow.

**Methods:**

We developed tags that were attached to relevant assets paired with Bluetooth Low Energy sensor beacons, which served as the basis of the asset tracking system. Problems with the system were identified during implementation and operation, and the feasibility of introducing the system was evaluated via a satisfaction survey completed by end users after 3 months of use.

**Results:**

The results showed that 117 nurses who had used the asset tracking system for 3 months were moderately satisfied (2.7 to 3.4 out of 5) with the system, rated it as helpful, and were willing to continue using it. In addition, we identified 4 factors (end users, target assets, tracking area, and type of sensor) that should be considered in the development of asset tracking systems, and 4 issues pertaining to usability (the active tag design, technical limitations, solution functions, and operational support).

**Conclusions:**

The successful introduction of asset tracking systems based on real-time location in hospitals requires the selection of clear targets (e.g., users and assets) via analysis of the user environment and implementation of appropriate technical improvements in the system as required (e.g., miniaturization of the tag size and improvement of the sensing accuracy).

## Background

The appropriate placement and supply of commodities in hospitals not only improves the quality of patient treatment but also influences the outcomes of treatment in emergency situations. Therefore, commodity management could be considered an exceedingly important task that should not be neglected by hospitals [1–4]. Nurses are generally tasked with the management of commodities in medical sites, and when their shifts end, they transfer responsibility for the equipment in their departments and wards to nurses on the next shift. Therefore, the effective management of commodities and a reduction in the time required to transfer them between nurses would not only increase the time available to nurses for the provision of patient care but also improve care quality [5–9].

One method of improving commodity management involves real-time location systems (RTLSs), which are used to manage assets efficiently in other industries. Interest in the introduction of these systems to the healthcare field is increasing, [10] albeit gradually [5, 11]. In healthcare, RTLS solutions are used mainly to track medical staff, patients, and assets. The introduction of these solutions has been shown to provide hospitals with certain benefits [5, 7, 10, 12–16] including cost reduction, improvements in medical care quality and work processes, and increased patient satisfaction.

Recently, the healthcare sector has adopted the barcode, radio-frequency identification (RFID), WiFi, and Bluetooth sensing technology in the development of tracking systems. Barcode technology, in particular, has been widely adopted by nurses to reduce medication administration errors and the related costs and to improve patient safety [17]. Furthermore, previous studies have shown that these systems are effective in reducing costs, improving the quality of medical services, enhancing work processes, and boosting patient satisfaction, by tracking medical professionals and patients [6, 8, 10, 12–16, 18, 19]. RTLS technology is based mainly on the use of WiFi and RFID, although some research has examined whether Bluetooth could be used as the basis of RTLSs [5, 6, 20]. Researchers in the healthcare field have demonstrated strong interest in localization systems; however, these systems are still in their infancy. Given the differences between sensors in terms of trackable distance, cost, and accuracy, people should consider the pros and cons of sensors to ensure selection of the most appropriate product [21]. In addition, the user interface, security, and effects of interference need to be considered [22, 23]. Therefore, system users should share their experiences of implementation with others to aid medical institutions that are willing to adopt the technology in developing the most suitable systems [6, 11].

Therefore, this study aimed to share considerations and insights and to provide information regarding the adoption of an RTLS in a medical environment, obtained via the implementation and operation of a real-time Bluetooth Low Energy (BLE)/WiFi-based asset tracking system in a tertiary general university hospital.

## Methods

### Development of the BLE/WiFi-based asset tracking system

The development of the asset tracking system initially involved obtaining the definition of the asset management process by various medical professionals including a medical doctor, 4 nurses, a medical informatics professor, and 2 staff members from the asset management department at the tertiary general university hospital to which the authors are affiliated. Thirty developers ultimately participated in the development process, which took place over the course of 13 months, from November 2013 to November 2014. For approximately 2 months thereafter, we stabilized the system and performed tests to improve the software used to develop the BLE beacon, active tags, and web-based asset management application during the 13-month period.

**Fig. 1:**
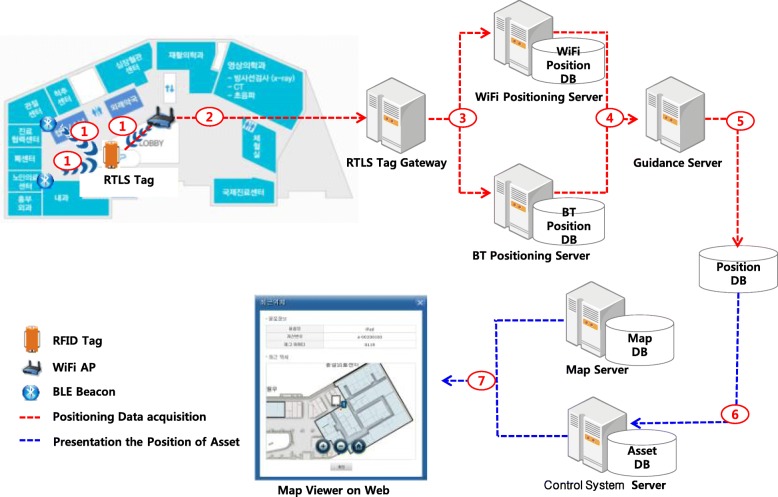
Overall architecture of the asset tracking system

The asset tracking system tracked the locations of assets throughout every ward in the hospital using the existing WiFi infrastructure; however, we used separate BLE beacons to provide location measurements with greater accuracy in the emergency room and the storage areas of the wards. The location of each asset and the status of the active tag battery could be checked using the hospital information system, into which the asset management system was integrated. Figure 1 shows the overall architecture of the BLE/WiFi-based asset tracking system.

**Fig. 2:**
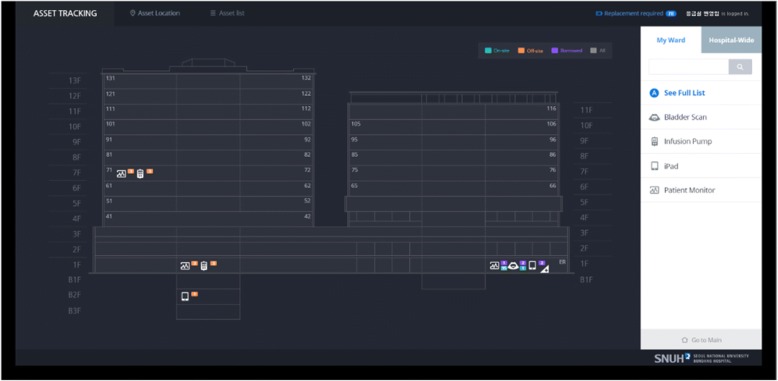
Interface of the asset tracking system for users (Nurses)

The asset management application consisted of 2 applications: 1 for users (i.e., nurses) and 1 for system administrators. In the user application, the types and positions of the assets on each floor were depicted as a bird’s eye view of the entire hospital, with the detailed locations marked clearly on a floor plan (see Fig. 2). The user application also provided detailed location information according to the asset status (i.e., onsite, offsite, or borrowed) for each department. In contrast, the administrator application provided information regarding the registration and management of target assets, the assets’ most recent locations and location histories, whether assets were onsite or offsite, and battery power levels for the tags. In addition, administrators could check the status of specific assets and all assets in a registered department using the search filter.

### Active tags and BLE beacons

The active tags and BLE beacons were designed in consideration of the characteristics of the medical environment and RTLS-based tracking system. Because the active tags were used to track highly mobile assets, they were battery operated and able to transmit their own location information based on data received from the beacon. Battery capacity was prioritized during the production stage, with the aim of maximizing the convenience of the tags. We ultimately selected tags that were 66 × 40 × 25 mm in size and required 2 AA batteries (3000 mAh, 3.0 V). We attached tags to a total of 400 instruments, including 4 iPads and 21 oxygen holders in the emergency room, 200 infusion pumps in the emergency room and 3 intensive care units (ICUs), and 160 patient monitors and 15 bladder scanners in all departments. The tag locations were selected to minimize interference with the equipment and prioritized according to improvements in nursing workflow and relocation frequency.

The beacons relied on BLE, which is a low-power, low-cost solution that has been used widely in recent years. They operated with replaceable batteries to obviate the need for separate power supplies. The beacons were 120 × 120 × 30 mm in size, with an estimated battery life expectancy of a year. In total, 98 BLE beacons were created; of these, 53, 31, 4, and 9 were positioned in the emergency room, the wards, the ICUs, and other hospital areas, respectively. The beacons were installed on the ceiling of each location.

### Setting asset tracking periods

The continuous transmission and reception of location information is essential for real-time location confirmation. However, because the active tags were battery powered and attached to mobile equipment, real-time transmission of the location information caused a battery drainage problem. Accordingly, in consideration of the battery life, we set each tag to transmit location information 16 times per day within a different period, mainly during the nurses’ work shifts, to avoid the burden of frequent battery replacement. Specifically, tag location signals were emitted at 30-min intervals throughout the nurses’ work shifts and at 2-h intervals during other hours. This setting was determined after consulting nurses who worked in the field and considering the system’s operational efficiency according to the battery performance of the tags. The 16 specific times per day were finally set as 06:00, 06:30, 07:00, 09:00, 11:00, 13:00, 13:30, 14:00, 14:30, 15:00, 17:00, 19:00, 21:00, 21:30, 22:00, and 23:00. Tracking 16 times per day was a rule that we implemented upon introducing the system, but the tracking intervals can be modified to provide optimal settings that reflect the users’ needs.

### Satisfaction questionnaire for the end users

**Fig. 3:**
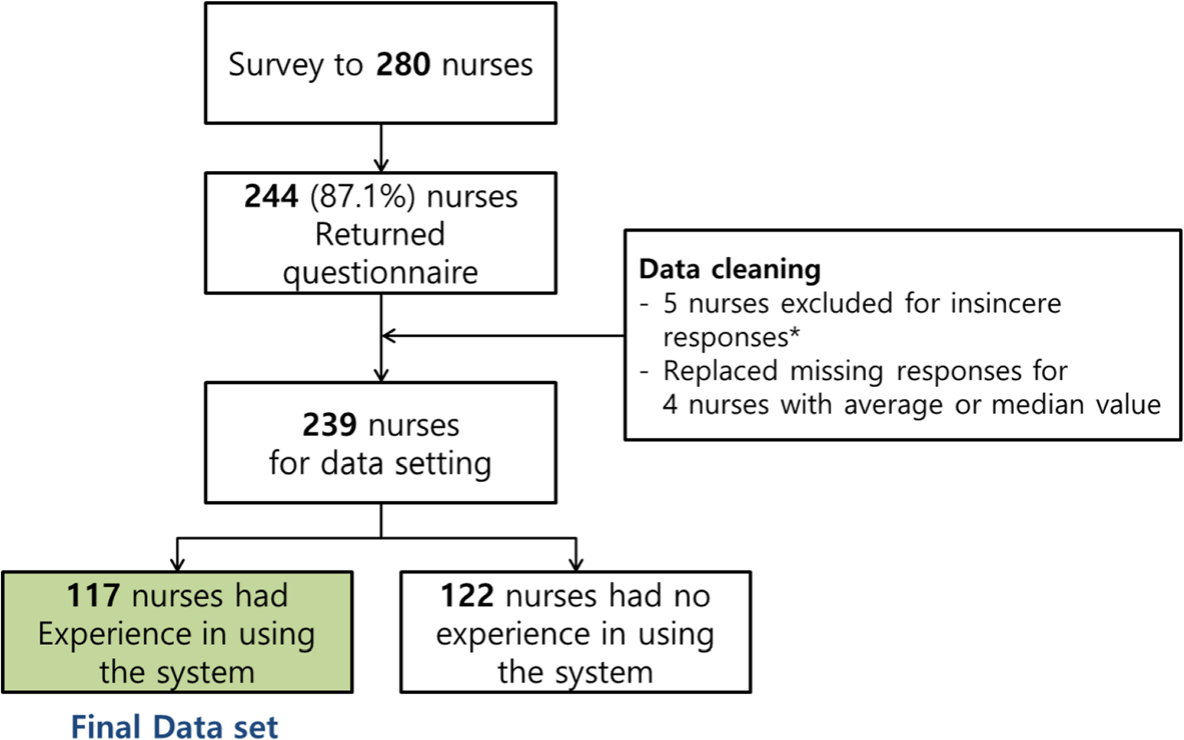
Participant flow in the user satisfaction survey. * The insincere response means respondents having same answers for all questionnaires

Following the development of the asset tracking system, a survey was conducted to examine the perceived quality and usability of the pilot system and to determine the end users’ overall satisfaction over the course of 2 months starting from December 2014. Nurses were the end users for the asset tracking system. Although the system was installed in the emergency room and all wards and ICUs, the self-report satisfaction survey was conducted only in the 3 wards with the highest numbers of users, 3 ICUs (i.e., surgical, internal medicine, and neonatal), and the emergency room. In total, 280 nurses completed the survey over the course of a month (February 16 to March 16, 2015). We received 244 anonymous responses (response rate: 87.1%), and the final dataset contained data for 117 nurses following data cleansing and the exclusion of data for nurses who had not used the system (Fig. 3).

The survey questionnaire was developed based on the Computer System Usability Questionnaire to assess the overall system, and the Questionnaire for User Interface Satisfaction to assess the user experience. The questionnaire consisted of 17 items divided between 5 categories, with responses provided on a 5-point scale. The questionnaire data were analyzed via frequency analysis and the Kruskal-Wallis test, using SAS 9.3 (SAS Institute, Cary, NC, USA) to determine whether the results were affected by the participants’ demographic characteristics.

## Results

### End-user satisfaction

The end user participants who completed the satisfaction questionnaire were primarily women, which is probably due to the nature of the nursing profession, and a large proportion were general duty nurses. In addition, the participants were evenly distributed between departments (i.e., wards, ICUs, and the emergency room) and were primarily in their 20s and 30s (Table 1).

**Table 1:** Participants’ demographic characteristics

Classification		*n*	%
Sex	Male	4	3.4
	Female	113	96.6
Position	Head nurse	8	6.8
	Nurse	109	93.2
Department	Ward	32	27.4
	Intensive care unit	45	38.5
	Emergency room	40	34.2
Age group	20s	59	50.4
	30s	52	44.4
	40s	6	5.1
Work experience	< 1 year	28	23.9
	1–3 years	38	32.5
	3–5 years	12	10.3
	> 5 years	39	33.3
Total		117	100.0

The participants’ mean satisfaction ratings for the 17 items ranged from 2.7 (for the tag size) to 3.4 (for the provision of the information required for nursing work, the need for the system, and intention regarding its future use).

As shown in Table 2, the ICU nurses generally exhibited higher levels of satisfaction than the emergency room nurses. In addition, statistically significant differences were observed for all items in the expectancy effects category, which pertained to the system’s expected effects with constant use in the future, and for 3 of the 4 items in the information quality category, which reflected the accuracy and display of information. In other words, while the nurses’ acceptance of the system’s practical functions differed between departments, they gave similar responses regarding whether the asset tracking system provided functions that were necessary on the ground, and they intended to use the system.

**Table 2:** Comparison of the mean satisfaction ratings according to the department (Intensive Care Unit, Emergency Room, and Ward)

Survey items			*M* (*SD*)				*P*
			Total	ER	ICU	Ward	
Information quality	1	Does the asset tracking system provide the information necessary to perform nursing work?	3.4 (0.785)	3.3 (0.716)	3.5 (0.661)	3.3 (0.991)	0.201
	2	Is the information provided by the asset tracking system accurate?	2.9 (0.928)	2.4 (0.781)	3.3 (0.793)	2.9 (1.014)	< .0001*
	3	Is the information provided by the asset tracking system expressed using appropriate terminology and in an appropriate format?	2.9 (0.844)	2.6 (1.005)	3.2 (0.712)	2.9 (0.641)	0.006*
	4	Does the asset tracking system provide up-to-date information appropriate for the current situation?	2.9 (0.908)	2.6 (0.868)	3.3 (0.733)	2.8 (1.019)	0.002*
System quality	1	Is the asset tracking system easy to use?	3.1 (0.902)	3.0 (0.974)	3.2 (0.773)	3.1 (0.976)	0.297
	2	Are you satisfied with the speed of the asset tracking system?	3.0 (0.904)	2.6 (0.838)	3.2 (0.735)	3.0 (1.078)	0.010*
	3	Do you need a separate user manual for the asset tracking system?	3.6 (0.993)	3.5 (1.132)	3.7 (0.905)	3.6 (0.948)	0.824
	4	Are you satisfied with using the asset tracking system through the electronic medical record?	3.2 (0.886)	3.1 (0.841)	3.3 (0.879)	3.2 (0.954)	0.371
Active tag	1	Are you satisfied with the size of the tag affixed to the device?	2.7 (0.992)	2.6 (0.979)	2.7 (0.953)	2.9 (1.070)	0.712
	2	You can replace the battery by pushing open the top part of the tag cover. Do you think this method of opening and closing the tag is appropriate?	3.0 (0.895)	2.7 (0.905)	2.9 (0.915)	3.3 (0.745)	0.014*
Expectancy effects	1	Is the asset tracking system more helpful for asset management than the previous method (i.e., manual management)?	3.2 (0.928)	2.9 (0.810)	3.5 (0.894)	3.4 (1.008)	0.007*
	2	Is the asset tracking system more helpful for transferring the nursing instruments than the previous method (manual management)?	3.2 (0.952)	2.9 (0.778)	3.4 (0.957)	3.3 (1.085)	0.033*
	3	Is the asset tracking system more helpful for locating mobile equipment than the previous method (manual management)?	3.3 (0.972)	2.8 (0.813)	3.5 (0.894)	3.5 (1.078)	0.001*
	4	Do you think the equipment will be used more efficiently with the asset tracking system than with the previous method (manual management)?	3.3 (0.927)	3.0 (0.832)	3.5 (0.894)	3.5 (0.983)	0.017*
Overall satisfaction	1	Are you satisfied with the overall use of the asset tracking system?	3.1 (0.834)	2.7 (0.716)	3.3 (0.780)	3.3 (0.902)	0.002*
	2	Do you need the asset tracking system for your nursing work?	3.4 (0.884)	3.1 (0.883)	3.5 (0.815)	3.4 (0.946)	0.083
	3	Do you intend to continue using the asset tracking system?	3.4 (0.811)	3.2 (0.747)	3.5 (0.815)	3.6 (0.833)	0.068

*p < .05; comparisons were performed between 3 departments. *ER* emergency room, *ICU* intensive care unit

Table 3 shows the participants’ other subjective opinions (provided via free text in the questionnaire) of the ways in which the asset tracking system could be improved based on their 2-month pilot use. These opinions were classified according to whether they pertained to location accuracy, the tag, asset tracking, or user education. The most frequently observed comments concerned location accuracy in the system. In addition, we classified the content of the categories into subcategories and found that comments regarding expansion of the range of target assets for tracking were expressed frequently. Issues regarding the size of the active tag were also noted.

**Table 3:** Participants’ subjective opinions

Major classification	*n*	Sub-classification	*n*	Opinion
Locating/positioning	30	Detailed locations	7	I wish that the system showed more detailed locations in the ward
		Location accuracy	18	The item is not in the location indicated The location information is unreliable Location errors occur depending on the strength of the WiFi signal
		Tracking period	5	Real-time location identification would be nice
Active tag	18	Tag loss	7	The tags fall off the assets due to weak adhesion, and having to find the lost tags is burdensome
		Tag size	9	I wish the tag was smaller
		Battery cover	1	The battery cover falls off easily
		Battery life	1	The battery drains too quickly
Target assets	21	Tracking additional assets	10	I wish we could add tags to other equipment I wish that all the equipment in the asset categories (e.g., patient monitors) was included in the system^a^
		Asset identification method	11	In addition to the asset identifier being automatically assigned to each asset for the system to recognize, a manual method of managing asset identifiers would be nice
Education	4	Need for user education	4	An explanation of the purpose and use of the system is needed

Comments were provided via free text in the questionnaire

^a^Some assets (e.g., patient monitors) used by some departments were not tracked in the study

## Discussion

The following discussion considers the lessons that were learned during the introduction and pilot operation of the BLE/WiFi-based asset tracking system, based on the end users’ opinions.

### Scope of asset tracking

Medical institutions that intend to develop and introduce RTLSs are required to define the end users, target assets, tracking areas, and sensor types via thorough discussion prior to implementation [14, 20]. In the present study, nurses were selected as the end users of the system. Most nurses in South Korea are responsible for a higher number of patients relative to nurses in other advanced countries; nevertheless, they undertake various duties in addition to patient care. This additional work includes the management of assets between work shifts in order to ensure continuous care. A considerable amount of time is spent performing these duties. For example, in the present study, nurses managed 44 assets in 11 categories on the wards, 118 assets in 11 categories in the ICUs, and 265 assets in 33 categories in the emergency room. Furthermore, nurses are required to confirm the locations of all assets managed by their departments during handover to nurses on other shifts [14, 20].

**Fig. 4:**
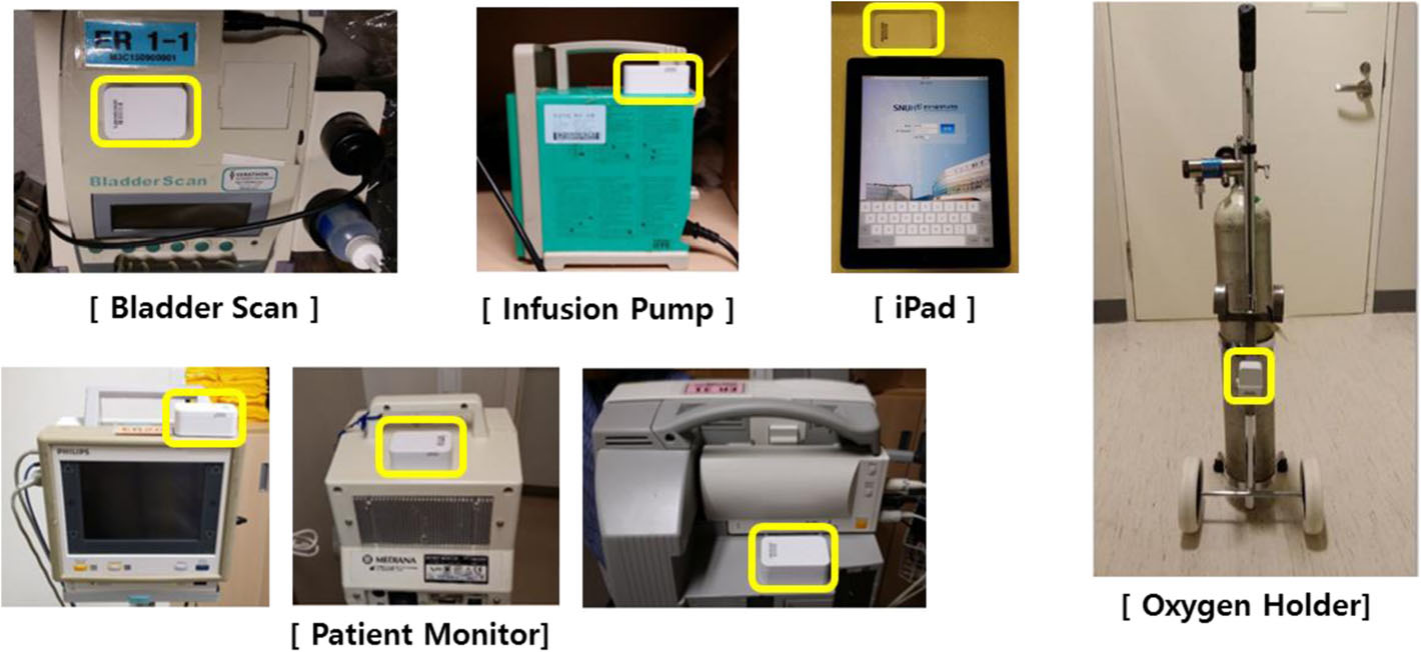
Five types of asset targeted for tracking. Locations at which the active tags were attached to the 5 types of equipment selected as asset tracking targets

The selection of the target assets for the pilot system’s implementation involved the creation of a list of all types of equipment managed by the nurses, which resulted in the selection of the most meaningful 5 assets (Fig. 4). Because of their high levels of mobility between departments, tracking the locations of these instruments was expected to increase nursing efficiency. The end-user satisfaction survey indicated that the asset tracking system was more helpful in managing/transferring/locating the assets than the manual management, with a satisfaction score that was above average. Considering the limited technology and limited tracking items used during the pilot, it seems that nursing work efficiency will be improved with the wider adoption of the system. Some examples of the practical use by the nurses in this study are as follows.

*“In the ward, it is not easy to trace the movement of patients who need various tests (MRI, CT, etc.), many procedures (emergency hemodialysis, angiography, etc.), and consultation. However, using the system, we were able to easily identify and collect the assets that the patient needed.”*

*“When it is urgent to inject an emergency patient with many medications, such as fluid, dopamine, dobutamine, Lasix, and albumin through an infusion pump, an asset tracking system can be used to quickly locate available devices. As such, medical staff can provide intervention to patients more quickly.”*

*“In the past, we had to check all of the assets we were managing, including the quantity and the location of the assets at the end of the shift. However, after using the asset tracking system, we can check the location and quantity at once by looking at the dashboard, which can save a lot of time.”*

The main reasons for equipment to be excluded were as follows: it was not needed by the end users, the tag influenced the usability of the asset, poor return on investment, and it did not fulfill the criteria for the tracking area (Table 4).

**Table 4:** Reasons for excluding assets from the real-time tracking

Asset	Reason for exclusion
Patient tracking	Little need for tracking at the site (e.g., psychiatric patients, neonates)
Laryngoscopy handle	Reduces the usability of intubation devices (i.e., it is difficult to affix the tag because it could hinder visibility) Difficulty in managing intubation devices (e.g., disinfection following each use)
Sandbag and ice pack	While necessary, the return on investment is low (i.e., the tag cost is high relative to the unit cost for these items)
Operating room items	The mobility of expensive medical instruments in the operating room is markedly low, so there is little need for tracking
Wheelchairs	While necessary, they were excluded because they are not for indoor use only (e.g., they are used outdoors frequently at medical institutions)

### Location vs. positioning

RTLSs use both access points (APs) and beacons, which are typically placed indoors, to ensure tracking accuracy. In the present study, locations were tracked using an existing WiFi infrastructure that covered all of the hospital wards, while the location positioning of greater accuracy in the emergency and storage rooms was measured via BLE beacons. Because errors begin to occur at a distance of 10 m for WiFi or 5 m for BLE, it is necessary to choose an appropriate type of sensor for each target asset and area [21, 24].

The system had several technical limitations. For example, the most important aspect of asset tracking is the ability to determine the precise location of a piece of equipment. However, the sensors do not always provide accurate locations in indoor tracking due to interference from other radio waves. Indeed, in the present study, users occasionally reported that assets were located on a different floor or in a different room to that shown in the tracking application; this is likely to have occurred because of the AP signal strength. In addition, we noted a problem with the batteries in the tags. As mentioned earlier, batteries were used in the tags and beacons to ensure their portability; however, periodical battery replacement for the beacons (once per year) and tags (every 100 days) would be difficult because of the sheer number of assets. Therefore, with respect to the tags, it would be necessary to decide whether to miniaturize them for greater usability or fit them with large-capacity batteries for greater operational efficiency. The sensing period for location identification would also require adjustment. This could be achieved using either signals generated by the tags or call signals from the beacon and AP; however, the provision of real-time measurements would require the generation of signals by the tags every 5 to 10 s, which would be fatal for battery life. In the present study, signal generation was limited to 16 times per day and focused on the nurses’ shifts. However, because the emergency room typically requires frequent movement of equipment, the generation of location information only 16 times per day might have been insufficient. The fact that the number of assets managed by the emergency room was higher, relative to those managed by other departments, could also explain the low satisfaction levels observed in the study.

This configuration increased the difficulty in providing real-time identification of asset locations, which was inconvenient for the end users. The limitations concerning accuracy and battery life coincide with technical limitations noted in other studies [5, 11, 14, 20, 25] and are expected to improve gradually. However, accurate location positioning using BLE may not be easily solved due to the frequency hopping of the BLE beacon [26]. Therefore, each site will be required to develop solutions to these issues as they arise. In addition, given the recent expansion of services based on wireless communication, and considering the interference caused by each signal, a more suitable method should be used to track assets [22, 23, 27].

### Design of the active tags

We developed the BLE/WiFi-based tags because the target assets did not include a signal-sensing function. However, this increased the size of the tags, which reduced their adhesive power and led to the loss of some tags. This occurred because we ultimately selected non-rechargeable AA batteries during the production process to increase the duration of the battery replacement cycle (i.e., lithium polymer [26 × 35 × 15 mm] and alkaline or manganese [AA size of 66 × 40 × 25 mm] batteries last for 20 and 100 days, respectively), and to ensure greater management efficiency. Although tag loss disrupted the continuity of asset tracking and incurred ongoing costs, the prioritization of battery capacity over tag size was unavoidable considering the long-term management efficiency. Because of this inverse relationship between the efficiency of the tag size and management, the needs of the site and the organization’s policy standards should be established and reflected in the production and introduction of the active tags.

### System functionality and usability

The interface used in the asset tracking system was integrated with the hospital information system, which allowed the end user to use the system while checking patient records. However, after 3 months of pilot use, the end users required a function to modify the names of assets since we displayed the names that were managed by the logistics department with a read-only mode for end users. This function could be integrated into the interface easily and would probably increase user satisfaction. Notably, the emergency room nurses’ satisfaction levels were considerably lower than those of nurses at the other sites, despite the higher location accuracy in the emergency room, which contained 53 beacons, while each storage room in the ICUs and wards contained a single beacon. It seems that the emergency room nurses had a higher expectation and a higher need initially, resulting in low satisfaction due to the accuracy problem. Okoniewska et al. also suggested that the RTLS system should improve the accuracy of location tracking and visualization in response to the converging opinions of nurses [20]. Furthermore, regarding the patients’ satisfaction with the patient tracking system, Stubig et al. found that their satisfaction improved when the medical staff also used the location information compared to when their location information was only tracked [11].

### Education and operational support

Users reported that sufficient support would be needed to improve the approachability and efficiency of the system. Educational efforts and a larger management workforce would be major factors in providing this support [20]. In addition, it would be necessary to supply sufficient information regarding the system operation and to provide appropriate education to ensure its smooth use. In addition, workforce support is required to ensure timely battery replacement (for the beacon and tags). In the present study, end users were not required to replace the batteries themselves because it was unnecessary during the 3-month pilot period, and the study was conducted during the maintenance period of the system development. However, these issues will require consideration in future; therefore, end users should be provided with the necessary information regarding battery replacement [20].

### Limitations

The main limitations of the present study were that it was conducted in a single medical institution, and the study design excluded the general outpatient environment. In addition, the tracking method was limited to WiFi and Bluetooth, and only some of the assets were tracked, which did not allow maximization of the benefits of tracking the assets used by nurses. Future research should be conducted in consideration of the issues highlighted in the present study. Moreover, future studies should not only involve the collection of data regarding end users’ subjective experiences but also include usability tests and time-motion studies.

## Conclusions

The results of the study demonstrated that BLE**/**WiFi-based asset tracking systems are needed in medical organizations and that they are helpful for nursing work. However, several technical and managerial issues were identified and should be addressed to ensure effortless use of the system and improve user convenience. In addition, the development and introduction of an asset tracking system in consideration of the lessons learned from the present study is required.

## Abbreviations

AP: Access point
BLE: Bluetooth Low Energy
ICU: Intensive care unit
RFID: Radio-frequency identification
RTLS: Real-time location system
